# Anasarca Secondary to Protein-Losing Enteropathy Leading to the Diagnosis of a Gastrointestinal Neuroendocrine Tumor in a Young Female: A Case Report From an Indian Suburb

**DOI:** 10.7759/cureus.65745

**Published:** 2024-07-30

**Authors:** Prakash Shende, Subashini Vadivel, Sheetal Nandha Kishore, Dhairya Sanghani

**Affiliations:** 1 General Medicine, Dr. D. Y. Patil Medical College, Hospital and Research Centre, Pune, IND

**Keywords:** fecal alpha 1 antitrypsin, tumor marker, anasarca, protein-losing enteropathy, gastric neuroendocrine tumors

## Abstract

A 34-year-old woman presented with worsening generalized swelling and breathlessness for four months; physical examination showed pallor, diffuse anasarca, and bilateral crackles on respiratory auscultation. Laboratory investigations showed severe hypoproteinemia, fat malabsorption with fat-soluble vitamin deficiency, and significant protein loss in the stool. Imaging studies revealed pulmonary edema, ascites, bowel wall edema, and a duodenal polyp. Further evaluating the duodenal polyp, a grade two duodenal neuroendocrine tumor (NET) was identified. She was managed with subcutaneous octreotide and duodenal polypectomy, resulting in significant clinical improvement. This case highlights the importance of diagnosing and managing protein-losing enteropathy secondary to gastric neuroendocrine tumors.

## Introduction

Gastrointestinal neuroendocrine tumors (GNETs) are uncommon neoplasms, with an annual incidence ranging from one to two cases per 200,000 individuals. These tumors originate from neuroendocrine cells distributed throughout the gastrointestinal (GI) tract. Clinical manifestations vary depending on their location and hormone secretion profile. One less common but clinically significant presentation of GNETs is protein-losing enteropathy (PLE) [1]. PLE is the excessive loss of plasma proteins into the GI tract, leading to hypoproteinemia and subsequent edema. The pathophysiology of PLE in the context of GNETs involves mechanisms such as mucosal disruption, inflammation, or lymphangiectasia, which impair normal protein absorption and retention in the body [2]. Diagnostic workup often involves imaging modalities, endoscopic evaluation, and histopathological confirmation. Management strategies aim to control tumor growth, alleviate symptoms, and address nutritional deficiencies [2]. This case study explores the clinical features, diagnostic challenges, and current management strategies of PLE secondary to GNETs.

## Case presentation

A 34-year-old woman presented to the casualty department complaining of swelling all over her body and difficulty breathing. She was asymptomatic four months ago, then she noticed swelling in both legs, which progressively worsened to involve her entire body. In the past week, she was breathless at rest, which was not associated with chest pain or palpitations. Before the past week, she never had sleep disturbances secondary to breathlessness. There was no history of fever, weight loss, or decreased appetite. There were also no complaints of abdominal pain, nausea, vomiting, blood in stools, bowel, or bladder disturbances. She had no significant medical, family, or obstetric history.

On examination, she was found to have pallor and diffuse anasarca. There were no signs of icterus, cyanosis, clubbing, or lymphadenopathy. Her blood pressure was 100/60 mmHg, pulse rate was 112 beats per minute, oxygen saturation was 95% on four liters of oxygen, and respiratory rate was 30 breaths per minute.

Abdominal examination showed tense ascites without dilated veins or organomegaly. Respiratory system examination revealed bilateral crackles. The cardiovascular examination was normal, with no murmurs or added heart sounds, and the nervous system examination was within normal limits.

Initially, acute liver injury, acute kidney injury, or nephrotic syndrome was suspected. However, investigations revealed anemia due to deficiencies in iron and vitamin B12. Further examination uncovered protein deficiency, as well as deficiencies in calcium and vitamin D. Abnormalities in activated partial thromboplastin time (aPTT) and prothrombin time (PTINR) indicated a clotting factor deficiency associated with vitamin K deficiency. Additionally, fat malabsorption and resultant fat-soluble vitamin deficiencies were noted. Normal urine examination and normal liver function tests excluded the initial suspicions of liver or kidney dysfunction. The relevant lab values are presented in Table 1 and Table 2.

**Table 1:** Routine investigations LDH, lactate dehydrogenase; CRP, C reactive protein; ESR, erythrocyte sedimentation rate; PT-INR, prothrombin time international normalized ratio; HDL, high-density lipoprotein; LDL, low-density lipoprotein; aPTT, activated partial thromboplastin time; PT, prothrombin time

Investigation	Lab values	Reference values
Hemoglobin	9.5 g/dL	11.6-15 g/dL
Total leucocyte count	4800/μL	4000-10000/μL
Platelet count	312,000/μL	150,000-410,000/μL
Mean corpuscular volume	78.2 fL	78.2-97.9 fL
Total bilirubin	0.6 mg/dL	0.22-1.20 mg/dL
Direct bilirubin	0.3 mg/dL	<0.5 mg/dL
Indirect bilirubin	0.3 mg/dL	<1.0 mg/dL
Aspartate transaminase	42 U/L	<43 U/L
Alanine transaminase	44 U/L	<45 U/L
Alkaline phosphatase	104 U/L	35-104 U/L
Total protein	2.6 g/dL	6.4-8.3 g/dL
Albumin	0.9 g/dL	3.5-5.2 g/dL
Globulin	2.3 g/dL	2.3-3.5 g/dL
Serum sodium	136 mmol/L	136-145 mmol/L
Serum potassium	4.1 mmol/L	3.5-5.1 mmol/L
Urea	27 mg/dL	17-49 mg/dL
Creatinine	0.8 mg/dL	0.6-1.2 mg/dL
LDH	211 U/L	81-234 U/L
CRP	32 mg/dL	<10 mg/dL
Amylase	25 U/L	25-115 U/L
Lipase	32 U/L	73-393 U/L
ESR	5 mm/hr	<20 mm/hr
Serum iron	15 μg/dL	35-145 μg/dL
Total iron binding capacity	532 μg/dL	250-450 μg/dL
Transferrin saturation	18%	20-50%
Ferritin	4.0 ng/mL	4.6-204 ng/mL
Vitamin D	<3.5 ng/mL	20-50 ng/mL
Vitamin B12	123 pg/mL	160-900 pg/mL
Serum calcium	<6.0 mg/dL	8.6-10.2 mg/dL
Serum phosphorus	2.8 mg/dL	3.5-4.5 mg/dL
aPTT	42 sec	21.7-28.7 sec
PT	21.1 sec	10.8-13.1 sec
INR	1.8	0.85-1.15
Total cholesterol	48 mg/dL	<200 mg/dL
Triglycerides	42 mg/dL	<150 mg/dL
HDL	09 mg/dL	>40 mg/dL
LDL	32 mg/dL	<100 mg/dL
Intact parathyroid hormone	100 pg/mL	15-65 pg/mL
HbA1c	4.7%	<5.9%
24-hour urine protein	111 mg/24 hr	<149 mg/24 hr

**Table 2:** Additional investigation and its interpretation

Investigation	Inference
HIV (P24, HIV 1 Ab, HIV 2 Ab)/HCV Ab/HBsAg	Non-reactive
Urine routine	No proteinuria, no hematuria
Thyroid function test	Within normal limits
Ascitic fluid examination	Transudative with ADA <1
Pleural fluid examination	Transudative with ADA 4.5

2D-ECHO showed a normal left ventricular function (60%), no wall motion abnormality, and normal valvular function with mild pericardial effusion (non-tapable) (Video 1).

**Video 1:** 2D echocardiography of heart (parasternal short axis view) showing normal left ventricular function with a thin rim of pericardial effusion

**Figure 1:**
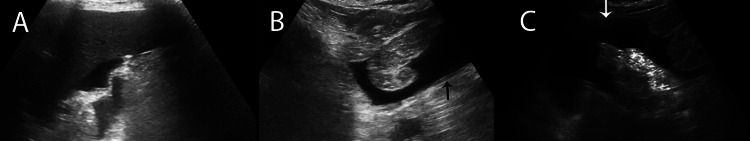
USG of the patient after paracentesis A: normal spleen; B (black arrow) and C (white arrow) show ascitic pockets

USG of the abdomen and pelvis revealed grade one fatty liver, normal kidney size, normal spleen, and moderate to gross ascites on presentation (Figure 1).

**Figure 2:**
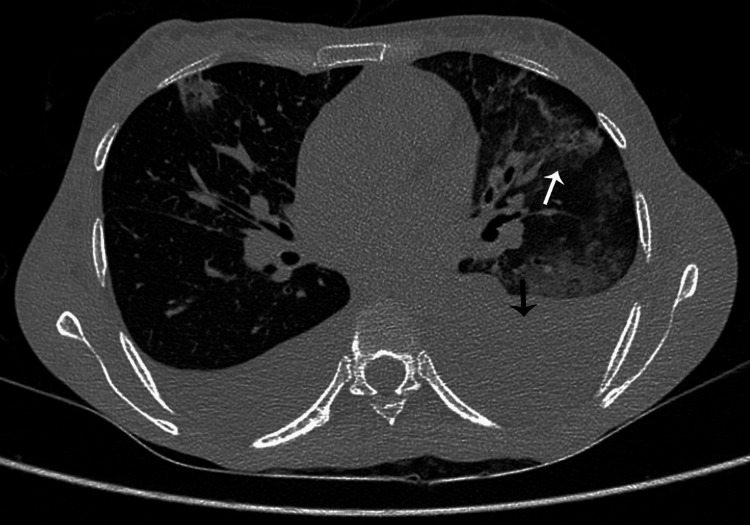
Bilateral pleural effusion (black arrow) and pulmonary edema (white arrow) noted in the HRCT of thorax

HRCT thorax showed diffuse ground-glass opacity over the anterior segment, posterolateral segments of the left lower lobe, and anteromedial segment of the right lower lobe of the lung, suggestive of pulmonary edema. Bilateral pleural effusion was noted (Figure 2).

**Figure 3:**
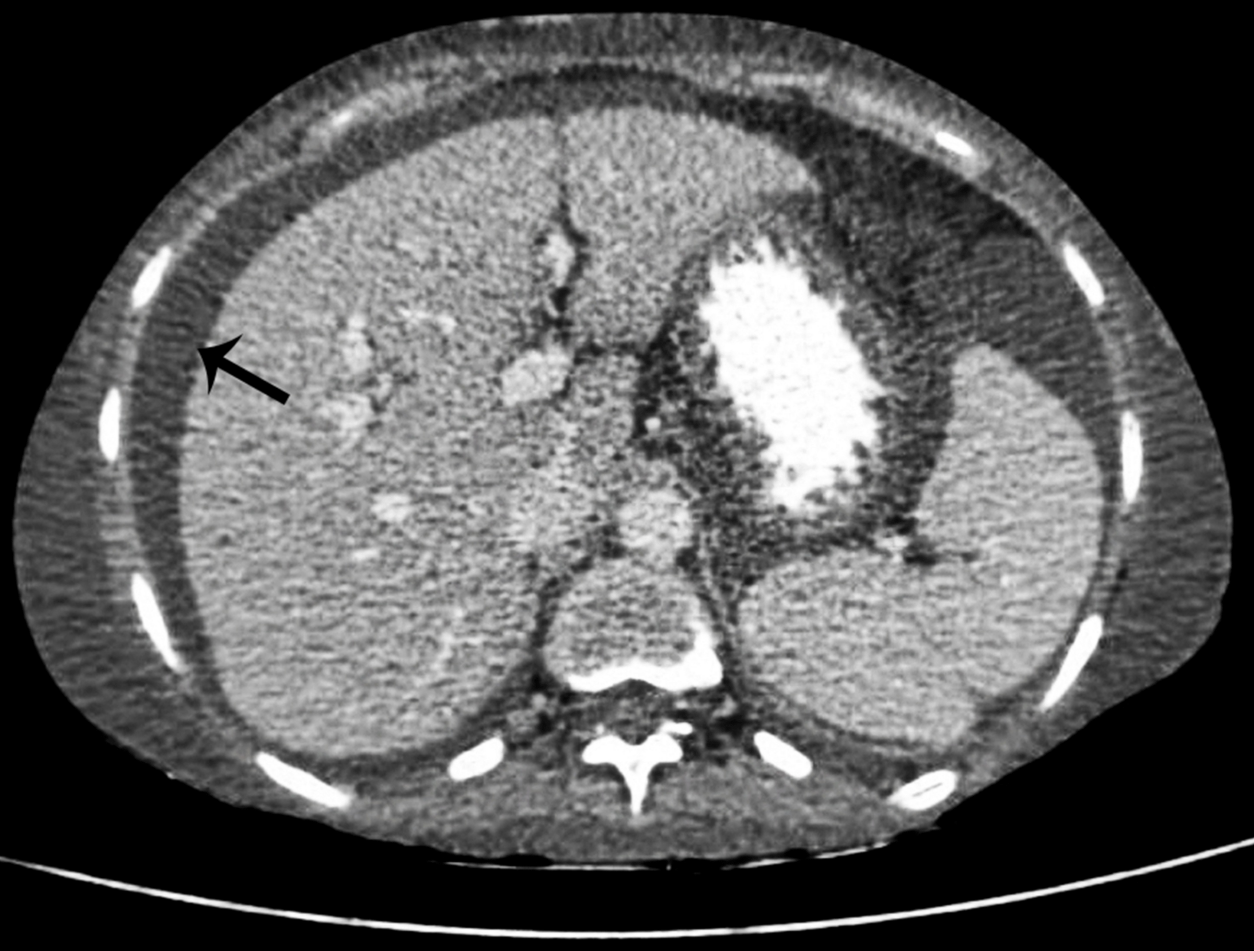
Black arrow showing ascites in the CECT of the abdomen

CECT of the abdomen and pelvis showed fatty liver, mild mucosal thickening in the bowel wall, normal kidney size, diffuse subcutaneous edema of the abdominal wall (anasarca), and gross ascites (Figure 3).

On day five, the patient's clinical status remained the same. Laboratory investigations showed no proteinuria, stool examination was normal (no fat globules), and 24-hour fecal alpha-1 antitrypsin (A1AT) levels indicated protein loss in the stool as shown in Table 3. The autoimmune panel and ANA profile were negative (Table 4).

**Table 3:** Lab investigation done to evaluate malabsorption Ig, immunoglobulin; A1AT, alpha-1 antitrypsin

Test	Result	Reference range
Serum tests		
Total IgG	374	700-1600 mg/dL
IgA	242	70-400 mg/dL
IgE	159	<150 IU/mL
Fecal tests		
Elastase	223	>200 µg/g
Calprotectin	43	50-200 µg/mg
A1AT clearance	57	<27 mL/24 hr

**Table 4:** Autoimmune panel and ANA profile LKM, liver kidney microsomal; ASMA, anti-smooth muscle antibody; SLA, soluble liver antigen

Test	Result
ANA by immunofluorescence	Negative
ANA by blotting	Negative
Anti-transglutaminase IgA	Negative
LKM antibody-1	Negative
ASMA test	Negative
SLA	Negative

**Figure 4:**
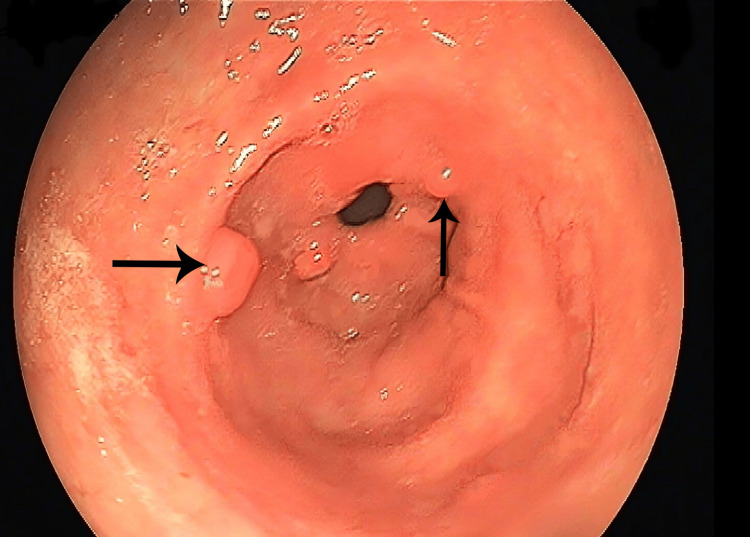
Upper GI endoscopy image The black arrow shows duodenal polyps. GI, gastrointestinal

An upper GI endoscopy revealed edematous stomach mucosa and two duodenal polyps measuring approximately 1 to 2 cm in size, with no ulcers or erosions observed (Figure 4). Deep enteroscopy showed edematous jejunal mucosa. The colonoscopy study indicated nodularity in the terminal ileum and edema from the cecum to the rectal mucosa. Fibroscan showed no evidence of liver fibrosis, and both the portal vein doppler and hepatic vein doppler studies were normal.

**Figure 5:**
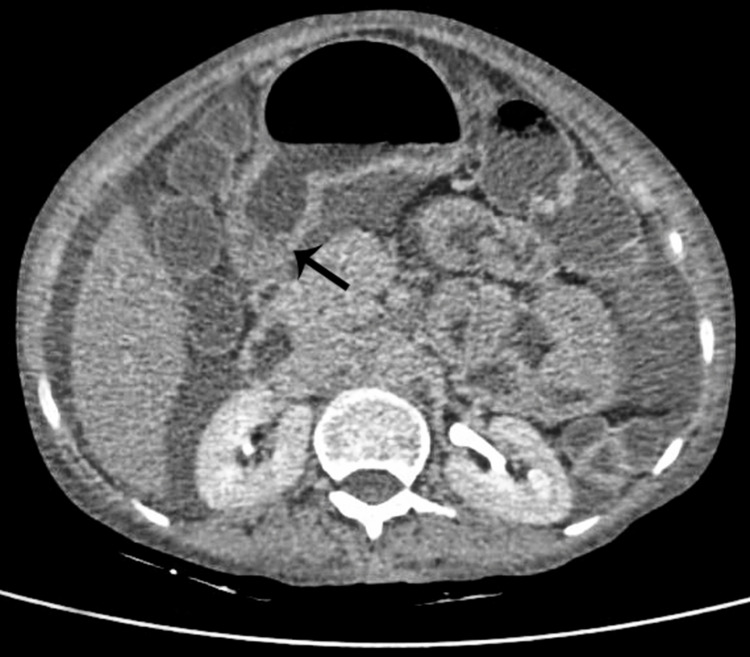
Duodenal wall thickening visible in CT enterography (black arrow)

CECT enterography shows mucosal thickening in the duodenum, jejunum, and stomach; the rest of the bowel appears normal in thickness (Figure 5).

On day 12, a duodenal biopsy report indicated normal mucosa, with lamina propria showing dense chronic inflammatory infiltrates. The muscular layer showed tumor cells composed of uniform cells arranged in sheets and small clusters, round to oval with moderate cytoplasm, with no evidence of atypical mitosis.

**Figure 6:**
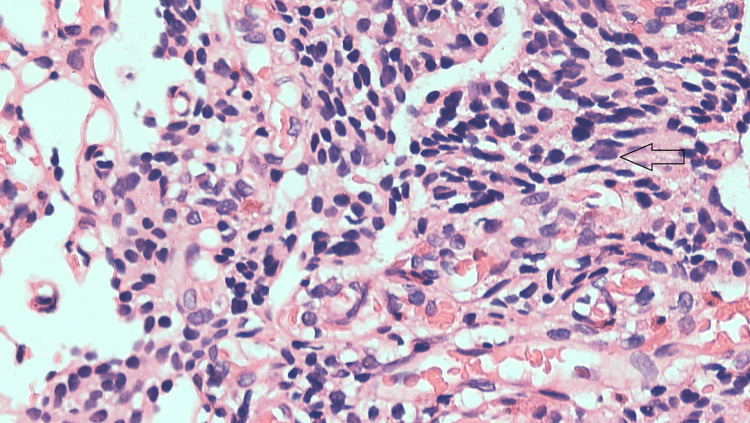
The black arrow mark shows oval tumor cells

**Figure 7:**
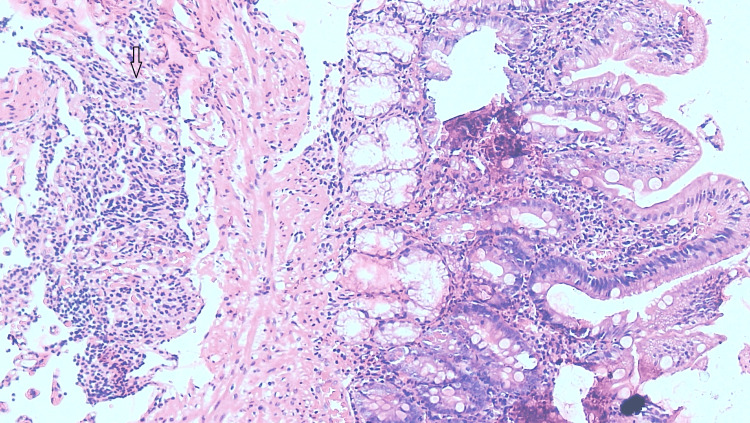
Tumor cells are seen in the muscularis propria layer Round-to-oval tumor cells (black arrow) in the muscularis propria layer.

Immunohistochemistry (IHC) markers showed synaptophysin cytoplasmic positivity, chromogranin positivity, and a Ki-67 index of 6-8%. Grade two neuroendocrine tumor (NET) of the duodenum was concluded (Figure 6 and Figure 7).

MEN syndrome workup and Ga-DOTATATE PET/CT were advised, but the patient declined further investigation due to financial constraints.

She was started on subcutaneous octreotide injections for long-term maintenance therapy, and a duodenal polypectomy was performed. After a few weeks, the patient showed significant improvement, and both anasarca and malabsorption resolved (Table 5).

**Table 5:** Comparison of the lab investigations on admission and the day of discharge aPTT, activated partial thromboplastin time; PT, prothrombin time; NR, international normalized ratio; HDL, high-density lipoprotein; LDL, low-density lipoprotein

Investigation	On admission	Day of discharge	Reference value
Hemoglobin	9.5 g/dL	11.2 g/dL	11.6-15 g/dL
Total protein	2.6 g/dL	5.8 g/dL	6.4-8.3 g/dL
Albumin	0.9 g/dL	3.1 g/dL	3.5-5.2 g/dL
Globulin	2.3 g/dL	2.7 g/dL	2.3-3.5 g/dL
Serum Iron	15 μg/dL	52 μg/dL	35-145 μg/dL
Total iron binding capacity	532 μg/dL	400 μg/dL	250-450 μg/dL
Transferrin saturation	18%	35%	20-50%
Ferritin	4.0 ng/mL	108 ng/mL	4.6-204 ng/mL
Vitamin D	<3.5 ng/mL	18 ng/mL	20-50 ng/mL
Vitamin B12	123 pg/mL	185 pg/mL	160-900 pg/mL
Serum calcium	<6.0 mg/dL	8.3 mg/dL	8.6-10.2 mg/dL
Serum phosphorus	2.8 mg/dL	3.8 mg/dL	3.5-4.5 mg/dL
aPTT	42 sec	29 sec	21.7-28.7 sec
PT	21.1 sec	12 sec	10.8-13.1 sec
INR	1.8	1.13	0.85-1.15
Total cholesterol	48 mg/dL	78 mg/dL	<200 mg/dL
Triglycerides	42 mg/dL	85 mg/dL	<150 mg/dL
HDL	9 mg/dL	38 mg/dL	>40 mg/dL
LDL	32 mg/dL	67 mg/dL	<100 mg/dL

## Discussion

Protein-losing enteropathy

PLE manifests as a symptom rather than a definitive diagnosis. Any patients presenting with low protein levels must be evaluated thoroughly for common causes like chronic liver diseases, severe malnutrition, and nephrotic syndrome before considering PLE. The protein loss in PLE occurs irrespective of the size of the protein molecule, leading to decreased serum levels of both albumin and globulins [3,4].

Pathophysiology

PLE arises when GI loss of protein exceeds the body's ability to compensate. The underlying causes can vary; important ones are discussed below in Table 6.

**Table 6:** Pathophysiology of PLE AIDS, acquired immunodeficiency syndrome; SLE, systemic lupus erythematosus; CHAPLE, complement hyperactivation, angiopathic thrombosis, and protein-losing enteropathy; PLE, protein-losing enteropathy; HKLLS, Hennekam lymphangiectasia-lymphedema syndrome; NTE, neuroendocrine tumor

Mechanisms causing protein loss	Conditions
A breach in the mucosal membrane causes malabsorption and protein loss	Infective etiologies such as gastroenteritis, ulcerative or erosive lesions in the stomach and duodenum, Zollinger-Ellison syndrome, and pseudomembranous colitis [3,4,5].
Inflammation or lesions on the mucosal surface decrease the surface area for absorption	Inflammatory bowel disease, celiac disease, tropical sprue, AIDS-associated enteritis, autoimmune diseases like SLE, food allergies, Ménétrier’s disease, eosinophilic gastroenteritis, amyloidosis, and graft-vs-host reaction [3,4,5,6].
Increased hydrostatic pressure or lymphatic obstruction of the splanchnic circulation	Right heart failure, Fontan procedure, heart failure with congestion, cirrhosis with portal hypertension, Budd-Chiari syndrome, lymphoma, mesenteric tuberculosis, and lymphangiectasia [3,7].
Mechanisms by which neoplasia causes PLE: It can be due to direct mass effect, increased peristalsis, decreased surface area of absorption, mucosal and submucosal inflammation, or as part of a paraneoplastic syndrome.	NTEs, neuroblastoma, Waldenström macroglobulinemia, Langerhans cell histiocytosis, Lynch syndrome, and familial adenomatous polyposis [1,4,5,6].
Congenital syndromes or gene mutations associated with defective lymphatic drainage result in PLE	HKLLS, Noonan syndrome, thanatophoric dysplasia, and CHAPLE disease [1].

Diagnosis

Diagnosing PLE requires extensive evaluation to identify the underlying cause. Laboratory tests, imaging studies, and endoscopic procedures are essential in guiding the diagnostic process. A crucial diagnostic step is demonstrating elevated fecal loss of A1AT [3,8].

A1AT clearance=(volume of the stool)×(stool A1AT)/(serum A1AT)

An elevated A1AT clearance (>27 mL/day) suggests GI protein loss, with approximately 80% sensitivity. Spot stool A1AT tests are less sensitive, while random stool A1AT levels with serum A1AT are practical for monitoring PLE treatment. When A1AT clearance is inconclusive, technetium 99m-labeled human serum albumin (HSA) scintigraphy can provide further diagnostic insights [8]. Imaging studies like CT or MRI may help in identifying the underlying cause, such as lymphatic obstruction or tumors.

Treatment

Treating the underlying cause is crucial. A protein-rich diet (2-3g/kg/day) is recommended, along with nutritional supplements to ensure adequate electrolytes, micronutrients, and vitamins. Regular monitoring of A1AT clearance or spot stool A1AT levels after initiating the treatment is recommended [3,4,6].

Gastric neuroendocrine tumors

GNETs are generally rare tumors; in the United States, the annual incidence is one to two cases per 200,000 people. These tumors originate from neuroendocrine cells of the pancreas, GI tract, and lungs and less commonly in the breast, prostate, thymus, and skin. They can manifest as well-differentiated tumors or they can progress to carcinomas, which are poorly differentiated [9].

Clinical presentation may vary from hormone-related symptoms from the tumor (functional NETs) or due to mass effect and metastasis. Common symptoms include flushing, diarrhea, abdominal pain, and symptoms of obstruction or bleeding [9,10].

Neuroendocrine cells produce functional hormones like serotonin, insulin, glucagon, and gastrin, depending on the specific subtype of the tumor. GNETs produce different hormones based on their cell of origin. For instance, duodenal NETs can present as gastrinomas, somatostatinomas, or paragangliomas. Gastrinomas are often associated with conditions like Zollinger-Ellison syndrome and multiple endocrine neoplasia (MEN 1) [9,10,11]. Hormones and tumor markers of GNETs are mentioned in Table 7.

**Table 7:** Common gastric neuroendocrine tumors and associated hormones EC, enterochromaffin cell, HTP, hydroxytryptophan, NSE, neuron-specific enolase, CgA, chromogranin A; HIAA, hydroxyindoleacetic acid; PG, prostaglandin

S.No	Tumor type	Tumor location	Hormones	Tumor marker
1	Atypical carcinoids	Foregut EC cells	5-HTP, histamine	NSE, serum CgA
2	Carcinoid	Mid- and hindgut EC cells	Serotonin, PG	Urinary 5-HIAA, serum CgA
3	Insulinoma	Beta cells of the pancreas	Insulin	Serum insulin, C-peptide
4	Gastrinoma	Gastrinoma triangle	Gastrin	Serum gastrin
5	Glucagonoma	Pancreatic alpha cells	Glucagon	Serum glucagon, serum pancreatic polypeptide
6	Somatostatinoma	Pancreatic delta cells	Somatostatin	Serum CgA, pancreatic polypeptide
7	VIPoma	Non-beta islet cells	Vasoactive intestinal peptide	Serum CgA
8	Pheochromocytoma	Adrenal gland	Catecholamines	Urinary or plasma metanephrine

Diagnosis

Tumor marker: Non-specific tumor markers include chromogranin A, pancreatic polypeptide, and neuron-specific enolase. Specific markers are serum gastrin, metanephrine, glucagon, and insulin, as mentioned in the Table 7 [10,11].

Histopathology: The NTE cells have oval nuclei with granular chromatin. Grading and differentiation determine the aggressiveness of the tumor. Two important features that decide the proliferation index of the cancer are the Ki67 index (a protein produced in large amounts during cell proliferation) and mitosis per 10 hpf (high power field) [11,12]. WHO classification of GNETs is enumerated in Table 8.

**Table 8:** Histopathological features and grading of NETs The 2022 WHO classification of gastric neuroendocrine tumors and NEC [12]. NETs, neuroendocrine tumors; NEC, neuroendocrine carcinoma

Grade	Description	Mitosis per 10 HPF	Ki67 index (%)	Prognosis
Grade 1	Well-differentiated, low-grade tumor with minimal mitotic activity.	<2	<3	Median survival up to 10 years
Grade 2	Well-differentiated, intermediate-grade tumor with moderate mitotic activity or higher Ki67 index.	2-20	3-20	Median survival around 5 years
Grade 3	High-grade tumor with significant mitotic activity or high Ki67 index.	>20	>20	Median survival is approximately 1 year
Small cell NEC	Highly aggressive neuroendocrine carcinoma with small cell morphology and very high mitotic activity.	>20	Often >70	Generally poor prognosis
Large cell NEC	Highly aggressive neuroendocrine carcinoma with large cell morphology and very high mitotic activity.	>20	Often >70	Generally poor prognosis

Imaging: For suspected NET, it is recommended to undergo MRI or CT screening. CT of the chest, abdomen, and pelvis aids in tumor staging. Somatostatin analogs such as radio-isotope indium (In)-octreotide offer higher sensitivity for well-differentiated tumors [11,13,14].

Gallium-DOTATATE-PET/CT is the gold standard for diagnosing and staging NET [13]. Endoscopy and colonoscopy are crucial for visualizing tumors and obtaining biopsies. Endoscopic ultrasound is the most sensitive method for diagnosing pancreatic NETs [11,13].

Treatment

Treatment strategies often vary according to the specific characteristics and needs of individual patients. Table 9 lists the available treatment options.

**Table 9:** Treatment strategy for GNETs The treatment regimen is determined based on the tumor's size, grade, differentiation, and stage. Somatostatin analogs are used to alleviate symptoms caused by hormones produced by the tumor [9-13]. PRRT, peptide receptor radionuclide therapy; HAE, hepatic artery embolization; SIRT, selective internal radiation therapy

Treatment approach	Indications	Specific treatment options
Surgical management	Localized, well-differentiated tumors	Local resection: For size <2 cm, localized tumors without signs of invasion in histology or PET/CT. Radical resection: For tumor size >2 cm or those invading nearby structures. Lymph node dissection: remove affected lymph nodes in locally aggressive tumors.
Medical management	Symptomatic control, non-operable or metastatic tumor	Somatostatin analogs: lanreotide or octreotide for symptom control and tumor stabilization. Targeted therapy: Everolimus (mTOR inhibitor) or sunitinib (tyrosine kinase inhibitor). Chemotherapy: etoposide and platinum-based agents for aggressive tumors.
Radiation therapy	Palliative treatment, localized symptom relief	External beam radiation: For local symptom control and palliation. PRRT: Radioisotopes like lutetium-177-DOTATATE for targeting tumors expressing somatostatin receptors.
Liver-directed therapy	Liver metastases	HAE or SIRT: For liver-dominant disease and symptom control.
Supportive care	Symptom management, nutritional support, pain control	Nutritional support: Addressing malabsorption and nutritional deficiencies. Pain management: Addressing tumor-related pain. Psychosocial support: For patients and caregivers.

## Conclusions

In conclusion, managing GNETs that present with PLE requires navigating complex diagnostic pathways due to their rare clinical presentation. A thorough diagnostic approach is essential, combining clinical suspicion with advanced imaging, endoscopic evaluation, and histopathological analysis. Once diagnosed, treatment focuses on tumor control, correcting nutritional deficiencies, and managing symptoms to improve patient well-being. Recent advancements in imaging and targeted therapies have enabled more precise diagnosis and treatment planning. However, ongoing research is crucial to refine diagnostic criteria, optimize treatments, and ultimately improve outcomes for individuals with GI NETs complicated by PLE.
